# Directional interactions between current and prior saccades

**DOI:** 10.3389/fnhum.2014.00872

**Published:** 2014-10-28

**Authors:** Stephanie A. H. Jones, Christopher D. Cowper-Smith, David A. Westwood

**Affiliations:** Action Lab, School of Health and Human Performance, Dalhousie UniversityHalifax, NS, Canada

**Keywords:** saccade latency, peripheral cue, central cue, random walk paradigm, sequential saccades

## Abstract

One way to explore how prior sensory and motor events impact eye movements is to ask someone to look to targets located about a central point, returning gaze to the central point after each eye movement. Concerned about the contribution of this return to center movement, Anderson et al. (2008) used a sequential saccade paradigm in which participants made a continuous series of saccades to peripheral targets that appeared to the left or right of the currently fixated location in a random sequence (the next eye movement began from the last target location). Examining the effects of previous saccades (*n−x*) on current saccade latency (*n*), they found that saccadic reaction times (RT) were reduced when the direction of the current saccade matched that of a preceding saccade (e.g., two left saccades), even when the two saccades in question were separated by multiple saccades in any direction. We examined if this pattern extends to conditions in which targets appear inside continuously marked locations that provide stable visual features (i.e., target “placeholders”) and when saccades are prompted by central arrows. Participants completed 3 conditions: peripheral targets (PT; continuous, sequential saccades to peripherally presented targets) without placeholders; PT with placeholders; and centrally presented arrows (CA; left or right pointing arrows at the currently fixated location instructing participants to saccade to the left or right). We found reduced saccadic RT when the immediately preceding saccade (*n−1*) was in the same (vs. opposite) direction in the PT without placeholders and CA conditions. This effect varied when considering the effect of the previous 2–5 (*n−x*) saccades on current saccade latency (*n*). The effects of previous eye movements on current saccade latency may be determined by multiple, time-varying mechanisms related to sensory (i.e., retinotopic location), motor (i.e., saccade direction), and environmental (i.e., persistent visual objects) factors.

## Introduction

The ability to direct our gaze to relevant stimuli (e.g., locations, objects, events) within the environment is an important part of the process through which we detect and perceive visual information and interact with the world around us. Spatial and temporal changes in eye movements observed in relation to previous sensory and/or motor events can provide insight into the underlying neural mechanisms associated with both perception and action. A common approach in this regard has been to elicit saccades to targets distributed about a point of central fixation which serves as the starting point for all trials and to which the gaze must return after each target-directed saccade (e.g., Taylor and Klein, 2000; Fecteau et al., 2004; Reuter et al., 2006; Cowper-Smith and Westwood, 2013; Cowper-Smith et al., 2013). However, it is possible that any effects observed while employing such methodology may be influenced (or even contingent upon) the task structure requiring the participant to return their gaze to a central fixation point, because of the predictability that it introduces. In effect, following each saccade to an eccentric location, there is a completely predictable spatial, and often temporal, return of attention (or gaze) to the central fixation position.

As a way to circumvent the requirement for a return of gaze to a central position between saccade events, Anderson et al. (2008) employed a *random walk* consecutive saccade paradigm in which three participants (two of which were authors) made a continuous series of saccades (200 per run; participants completed 60 or 120 runs for a total of 12,000 or 24,000 saccades) to targets that appeared to the left or right (1.4°) of the currently fixated location in a random sequence. Each successive target appeared a constant distance to the left or right of the currently fixated location on a random basis, such that any saccade could equally well be followed by a saccade in the same or opposite direction. No placeholders (persistently visual target representations that indicate to participants where targets might appear) were used to mark the location of possible targets, and the currently fixated target disappeared simultaneously with the appearance of the subsequent target. Anderson et al. (2008) analyzed saccadic reaction time as a function of current saccade (*n*) direction (left or right), but more importantly the direction of preceding saccades (*n−1, n−2, n−3* and so on; same or opposite direction). It is important to note that the random walk paradigm permits an analysis of the independent effect of any number of preceding saccades' directions because the directions of the intervening saccades are randomly determined.

The authors observed significantly reduced saccadic latencies when the immediately preceding saccade (*n−1*) was in the same (vs. opposite) direction (i.e., leftward saccade reaction times were reduced when preceded by a leftward rather than rightward saccade). We refer to this pattern as a “same direction benefit” (SDB), in reference to the reduction in saccadic reaction time that was observed when a preceding eye movement was made in the same direction as the current eye movement. The magnitude of the SDB observed at the *1-back* level (i.e., the reaction time of a saccade as a function of the relative direction of the immediately preceding saccade) ranged from 4 to 14 ms for the three participants in the study (c.f. Anderson et al., 2008, Figures 1 and 4 in particular). Interestingly, the results of their *1-back* analysis are also entirely consistent with the presence of the phenomenon of inhibition of return (IOR, the time-dependent slowing in participants' ability to orient to and process information in a previously attended and/or fixated location; Posner and Cohen, 1984) since consecutive saccades in opposite directions return the gaze to the most recently inspected locations and should result in longer reaction times.

Unlike paradigms requiring a return of gaze to a central location between each saccade, the random-walk paradigm permits an analysis of the reaction time of a particular saccade (*n*) as a function of the relative direction of any number of previous saccades (*n−1, n−2*, etc.). These effects are computed by averaging across all instances in the sequence when a saccade is preceded by an “n-back” saccade (i.e., a saccade preceding the current saccade) in the same or opposite direction; such averaging does not create systematic biases because the intervening saccades in each instance are in random directions. Interestingly, Anderson et al.'s result showed a significant but exponentially decreasing SDB for *n-back* levels greater than one: SDB, previous saccades in the *same* direction contributed to a significantly decreased saccadic latency, even if separated by as many as 5 saccades, regardless of the directions of the intervening eye movements.

This is an important result because it indicates that the SDB cannot be attributed to a simple location-based effect such as IOR. Only at the *n−1* level is it true that two saccades in opposite directions *necessarily* return the gaze to the most recently inspected location, and that two saccades in the same direction *necessarily* bring the gaze to a new location. For *n−2* and higher levels, there can be multiple intervening saccades in any direction so there is no systematic relationship between the relative directions of the two saccades in question and the locations from which those saccades were generated. Hence, any difference in saccadic reaction time due to the relative direction of the *n−2 or higher* saccade cannot be due to location-based effects. As such, Anderson et al. (2008) suggest that the previous-saccade effects they observe in the random walk paradigm are distinct from IOR and are driven by the similarity of the directions of the current and previous saccades, rather than the return of gaze to a previously inspected location. This is an important consideration for studies that use a return to a central fixation task structure, because in this case the slower “same location” trials consist of a series of three movements (L-R-L or R-L-R) for which the final saccade is necessarily in the opposite direction from the most recently completed eye movement. According to Anderson et al.'s interpretation, this sequence of saccades may result in a slower final saccade not because the gaze returns to an old location but rather because the final saccade is immediately preceded by an eye movement in the opposite direction.

Although Anderson et al. (2008) highlighted the relevance of their results “… in the real world… ” (p. 614) and suggested that their results might indicate “… [that] the neural centers responsible for directing our gaze—and consequently, our overt attention—have evolved to reflect the patterns of the real world environment.” (Anderson et al., 2008, p. 617), their initial use of the random walk paradigm lacks several characteristics associated with eye movements in the “real world.” First, Anderson et al. (2008) showed only one target on the screen at a time, which turned off when the next target appeared. In the real world, gaze is often directed from one object to another, both of which remain visible before, during, and after the eye movement. Indeed, many studies exploring the effects on saccades of prior sensory and motor events, particularly those examining IOR, leave placeholder markers in the possible target locations (to indicate to participants approximate areas in which targets may appear) and it has been reported that the magnitude of IOR [which is relevant to the *1-back* analysis employed by Anderson et al. (2008)] can be reduced or eliminated in the absence of target location placeholders or other stable or permanent objects (e.g., Klein, 1988; Klein and MacInnes, 1999; Birmingham and Pratt, 2005). Second, Anderson et al. (2008) employed peripheral onset targets to draw gaze (and attention) to subsequent target locations. While some stimuli in the real world appear as a peripheral change in luminance, oftentimes the target of an eye movement is determined by a subjective change in the salience of an object whose luminance remains constant (e.g., identifying a currently fixated stimulus as a non-target and moving the gaze to a different object in the visual array, or perhaps looking at an object to which a friend is pointing). From a mechanistic standpoint, peripheral onset targets confound sensory and motor processes; an eye movement to a peripheral target that appears to the left of the currently fixated location could exhibit a reduced latency because it shares the same location on the retina as a previous peripheral target or because the direction of the eye movement is the same as the previous saccade. If the SDB observed by Anderson et al. (2008) arises from motor rather than sensory processes (as they propose), then similar effects should be observed regardless of whether saccades are elicited using peripheral onset targets or endogenous target selection. Here, we have employed central arrows and peripheral onset targets to elicit saccadic eye movements.

Therefore, the purpose of the present study was to extend Anderson et al.'s (2008) random walk sequential saccade paradigm to a group design involving task characteristics more similar to real-world stimulus detection scenarios. Twenty-six participants completed the random walk sequential saccade paradigm in three blocked, randomly ordered, stimulus conditions: (1) peripheral targets without placeholders: a single target appeared simultaneously with the offset of the previously presented target (similar to the methods employed by Anderson et al., 2008); (2) peripheral targets with placeholders: all possible target locations remained visible on the computer screen throughout the entire sequence of saccades (i.e., placeholders); and (3) centrally presented arrow targets with placeholders: an arrow presented at the currently fixated location signaled the direction of the subsequent saccade and placeholders for all possible target locations remained visible on the computer screen for the entire block of saccades. To date, ours is only the second paper to employ the random walk paradigm to examine saccadic dependencies.

## Materials and methods

### Participants

Twenty-six undergraduate Psychology students (5 males) aged 17–28 years (Mean age = 20.34 ± 2.60 years) from Dalhousie University, Halifax, N.S. participated in the current study in exchange for partial course credit. All participants had normal or corrected-to-normal vision, were right-handed (self-reported) and had no history of visual, motor or neurological abnormalities (self-reported). This study was approved by the Dalhousie University Social Sciences and Humanities Research Ethics Board. Written informed consent was provided by all participants prior to their participation. All participants completed all three sequential saccade conditions in which they responded to peripherally or centrally signaled targets (described in more detail below) in a random order.

### Procedures

**Figure 1:**
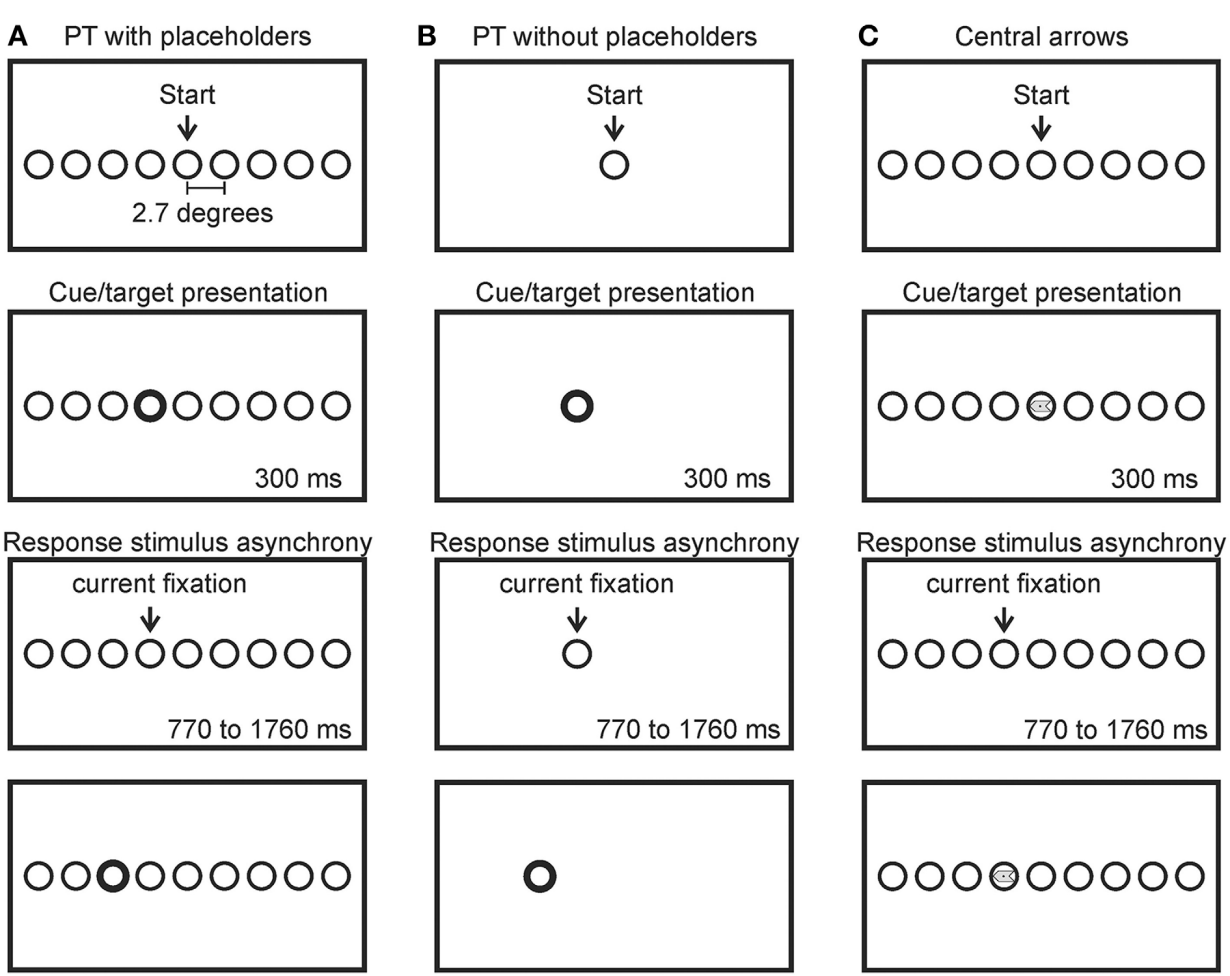
**Schematic (not to scale) of the random walk paradigm for the peripheral target (PT) with placeholders (A), peripheral target (PT) without placeholders (B) and central arrow conditions (C)**. Participants began by fixating the center circle (labeled “start”). A cue was then presented (bold outline of the target circle, the appearance of the target circle or a central arrow, respectively). Participants made a saccade to the cued target location where they maintained fixation for 770–1760 ms, after which another cue was presented signaling the subsequent target location to the left or right of the currently fixated location randomly. Only 9 of the 19 possible target locations are shown in the Figure.

Each participant completed a sequential saccade task (the random walk paradigm; Anderson et al., 2008) in three separate and randomly ordered conditions. A schematic sequence for each of the three conditions is presented in Figure 1 (not to scale). Participants were comfortably seated in a dimly lit room (consistent with Anderson et al., 2008), 75 cm in front of a 31” computer monitor (Tyco Electronics ©) on which all stimuli were presented using Experiment Builder® version 1.10.1 (SR Research Inc, Canada). Targets were circles (1.9° in diameter, black, presented on a white background) that could appear at 19 possible locations spaced 2.7° apart (center to center) along an imaginary horizontal line vertically centered on the computer screen (only a subset of the possible target locations is presented in Figure 1). Each condition began with the participant foveating the central target location (labeled “Start” in Figure 1). An infrared head-mounted eye tracking system (EyeLink II®; SR Research Inc, Canada) was used to record the position of the right eye at 250 Hz. The eye tracking system was calibrated before participants completed each condition. Participants were given a break in between conditions. Participants were instructed to move only their eyes in response to the onset of a target and were given an opportunity to practice in advance of their participation. The absence of head movement was ensured by the experimenter throughout the entire testing session.

#### Peripheral target conditions

In the peripheral target (PT) with placeholders condition (Figure 1A), all 19 possible target locations were continuously marked on the computer screen (i.e., as “placeholders”—distinguished from the target by a single feature, outline thickness) by circles matching the size of the target but with reduced line thickness (0.06 mm thick). The task began once the participant foveated the central circle (labeled “start” in Figure 1A). Subsequent targets were indicated by a “boldening” of the outline of the appropriate placeholder circle for 300 ms (0.16 mm thick). To create a random walk sequence of leftward and rightward saccades, targets were randomly presented at the placeholder one position to the left or right of the currently fixated location. Participants were instructed to saccade directly to the target circle and maintain fixation on that circle during the 770–1760 ms response-stimulus-interval until the next target appeared [response stimulus interval (RSI): the amount of time in between gaze reaching the region of interest of the target (the response) and the onset of the subsequent target]. The currently fixated circle was restored to its original line thickness (0.06 mm) coincident with the “boldening” of the subsequent target. Participants completed 204 saccades in this condition, comprised of 20 sequences of 10 saccades, separated by a self-timed drift correct (see below). Errors are defined below in Section Errors.

To limit fixational eye movements such as drift (e.g., Di Statsi et al., 2013), after every 10 saccades, the program would pause for the presentation of a drift correct [a dot positioned in the center of the central target location (i.e., “start”)]. As the beginning of a subsequent sequence of saccades after the drift correct was self-directed by the participant (through the press of a button or by asking the experimenter to advance the task), participants could use this time to rest their eyes if needed (they were instructed to limit their head and body movement during these breaks). Once ready to continue, participants began the next trial sequence with their gaze at the central target location (start); the program would not progress unless central target fixation was achieved. A description of the number of valid trial sequences for each n-back level is presented in the error section below and in Table 1.

**Table 1:** **Number of valid trial sequences for analysis, averaged across participants, for each condition and n-back level**.

	***n−1***	***n−2***	***n−3***	***n−4***	***n−5***
Peripheral targets without placeholders	131	102	78	59	44
Peripheral targets with placeholders	153	127	104	83	65
Central arrows	148	122	99	79	61
Average across conditions	144	117	94	74	57

In the PT without placeholders condition (Figure 1B), no placeholders were used to mark potential target locations. Circle targets simply appeared at new locations coincident with the offset of the circle at the currently fixated location (i.e., they appeared to “jump” from one location to another). All other spatial and temporal characteristics were identical to the PT with placeholders condition. This condition is most similar to that employed by Anderson et al. (2008).

#### Centrally presented arrows

In the central arrow condition (Figure 1C) all 19 possible target locations were continuously marked with circular placeholders (as in the PT with placeholders condition). Once the participant achieved fixation at the central location, the task would begin. A randomly selected leftward or rightward pointing arrow (1.9° long, 0.6° in width, equivalent area at the head and tail of the arrow) was presented for 300 ms, horizontally and vertically centered within the boundaries of the currently fixated placeholder. Participants were asked to saccade directly to the placeholder location immediately to the left or right of the current location, depending on the direction of the arrow (i.e., saccade to the placeholder location to the left if the arrow is pointing leftward and saccade to the placeholder location to the right if the arrow is pointing rightward). As in the other two conditions, participants were asked to maintain fixation within the placeholder until the next arrow was presented 770–1760 ms later. Consistent with the peripheral target conditions described above, participants completed 204 saccades in this condition, comprised of 20 sequences of 10 saccades, separated by a self-timed drift correct (as described above).

#### Errors

In all three conditions, if a response to a target took longer than 2000 ms, saccade endpoint was not within the region of interest of the target (i.e., within 0.6° from the perimeter of the target circle) or gaze deviated, drifted or was directed outside the region of interest of the current target in advance of a new target being presented, participants received an error message. A four second time penalty accompanied the error message and participants were instructed to return their gaze to the central target location (i.e., the start). A new target sequence began once the participants' gaze reached the central target location. In all conditions, the percentage of errors across all target events was used to assess the accuracy of saccades for each participant (Mean accuracy: PT without placeholders: 82% of all saccades; PT with placeholder: 89% of all saccades; CA with placeholder: 88% of all saccades). Target events associated with errors were excluded from further analyses (i.e., saccades associated with this target event were not considered as saccade *n*). Moreover, all *n-back* analyses (described later) were restricted to continuous sequences of target events free from errors or drift corrects, because errors and drift corrects interrupted the series of movements by requiring a return to the center target location. As such, RTs were computed for accurate saccades within error and drift correct free trial sequences in each of the three stimulus conditions. The average number of valid trial sequences for each n-back level for each condition are presented in Table 1.

## Analyses and hypotheses

### The effect of the immediately preceding saccade (n−1)

The primary analysis considered the latency of saccade *n* as a function of saccade *n* direction (left or right; this factor was included to take into account the possibility of asymmetries in saccade latency) and the relative direction of the immediately preceding saccade (saccade *n−1*: same or opposite). Each condition was analyzed separately. The *n−1* level was considered separately from all other *n* levels because, in this unique case, the presence of a SDB (i.e., for two consecutive leftward or rightward saccades) could be due to a directional interaction within the saccade motor control system, a location-based inhibition of return, or some combination of the two.

**Figure 2:**
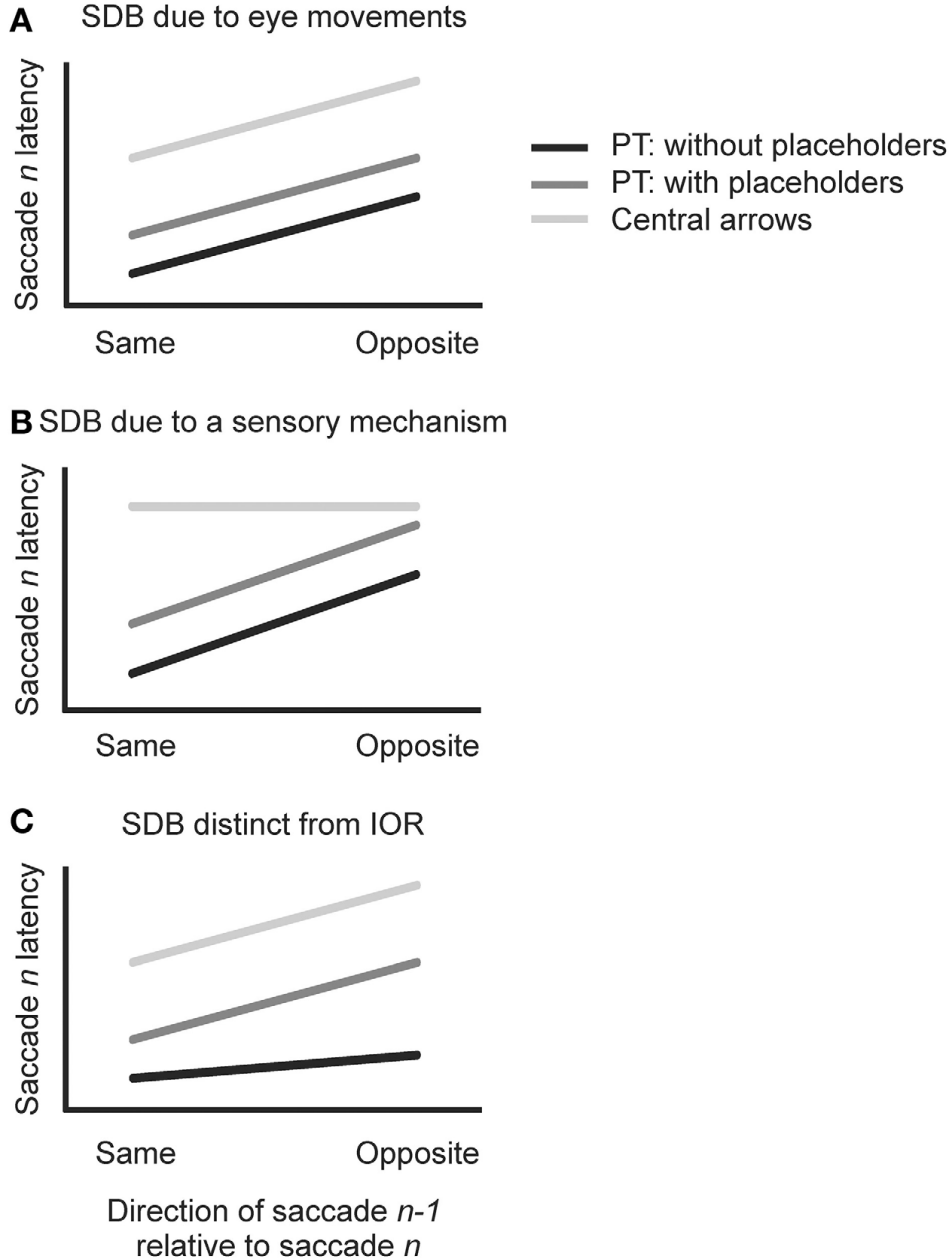
**Visual depiction of three possible outcomes of the *n−1* level analysis. (A)** For all conditions, saccade *n* latency is shorter when saccade *n−1* is in the same direction. **(B)** For PT without placeholders (black) and PT with placeholders (dark gray), saccade *n* latency is shorter when saccade *n−1* is in the same direction). No such difference is found between same and opposite conditions in the central arrow condition (light gray). **(C)** In all conditions, saccade n latencies are shorter when saccade *n−1* is in the same direction, but less so for PT without placeholders (black).

Figure 2 depicts three possible outcomes for the *n−1* level analysis. If, as Anderson et al. (2008) speculated, a SDB arises from the execution of eye movements *per se*, then similar SDB should be observed in all three conditions in the present study because all require sequential saccades (Figure 2A: for all conditions, saccadic *n* latency is shorter when the *n−1* saccade is in the same vs. opposite direction). If, instead, SDB arises from a sensory mechanism related to the different retinal locations stimulated by peripheral targets in the “opposite direction” as compared to “same direction” sequences, then SDB should be observed in the two peripheral target conditions but not in the central arrow condition (Figure 2B: for PT without placeholders and PT with placeholders only, saccade *n* latency is shorter when saccade *n−1* is in the same direction). If SDB at the *n−1* level is indeed distinct from the IOR phenomenon, then it should be observed whether or not possible target locations remain persistently visible during the task. In contrast, if SDB is related to IOR then it should be strongest in the conditions associated with placeholders and weakest in the single condition with no placeholders (Klein, 1988; Klein and MacInnes, 1999; Birmingham and Pratt, 2005) (Figure 2C: in all conditions, saccade *n* latencies are shorter when saccade *n−1* is in the same direction, but less so for PT without placeholders).

### The effect of preceding saccades (n−2 through n−5)

Secondary analyses considered the latency of saccade *n* as a function of saccade *n* direction (left or right), and the direction of the saccade completed 2, 3, 4, or 5 targets ago (i.e., *n−2* through *n−5*). Our choice to examine the effects previous saccades on current saccade latency up until 5 saccades previous was based on the primary finding reported by Anderson et al. (2008), that previous saccades in the *same* direction contributed to a significantly decreased saccadic latency, even if separated by as many as 5 saccades. Each condition was analyzed separately. Importantly, since target presentation is random, the two saccades of interest in these analyses (*n* and *n−x*) could be separated by saccades in any combination of directions (left or right). All possible random combinations of left and right intervening saccades are pooled together for analysis. Anderson et al.'s results showed a significant SDB for the *n−1* through *n−5* preceding saccades, so we expected to find something similar in the PT without placeholders condition (the task most similar to Anderson et al.). For the secondary *n-back* analyses, it was not clear what might be observed in the PT with placeholders and central arrow conditions.

### Data analysis

The dependent measure of interest, reaction time (RT) for saccade *n* was analyzed using a 2 (current saccade direction: left or right) × 2 (direction of the previous saccade of interest (*n−1, n−2, n−3, n−4, n−5* [separate analyses]: same and opposite) × 3 (condition: PT without placeholders, PT with placeholders, central arrow) repeated measures ANOVA. This analysis revealed significant interactions between condition and direction of the preceding saccade of interest at the *n−2* [*F*_(1.74, 43.62)_ = 3.75, *p* = 0.03], *n−4* [*F*_(1.96, 47.20)_ = 6.12, *p* = 0.005] and *n−5* levels [*F*_(1.75, 42.22)_ = 3.61, *p* = 0.04]. As such, subsequent analyses examined the effects of preceding saccade on current saccade latency for each condition separately using separate 2 (direction of saccade *n*: left and right) × 2 [direction of the preceding saccade of interest (*n−1, n−2, n−3, n−4, n−5*) (separate analyses): same and opposite] repeated measures ANOVAs (alpha = 0.05). As reaction times significantly differed as a function of the direction of saccade (i.e., whether the saccade was to the left or right) only in the PT with placeholders condition [*F*_(1, 25.08)_ = 4.63, *p* = 0.041] at the 1-back level, our results and discussion will focus on the effect of the relative direction of the *n-back* saccade on the reaction time of saccade *n*. Greenhouse-Geisser corrected *p*-values are reported. All analyses were conducted using IBM SPSS Statistics version 21 (IBM Corp. Released 2012. IBM SPSS Statistics for Windows, Version 21.0. Armonk, NY: IBM Corp.).

## Results

**Figure 3:**
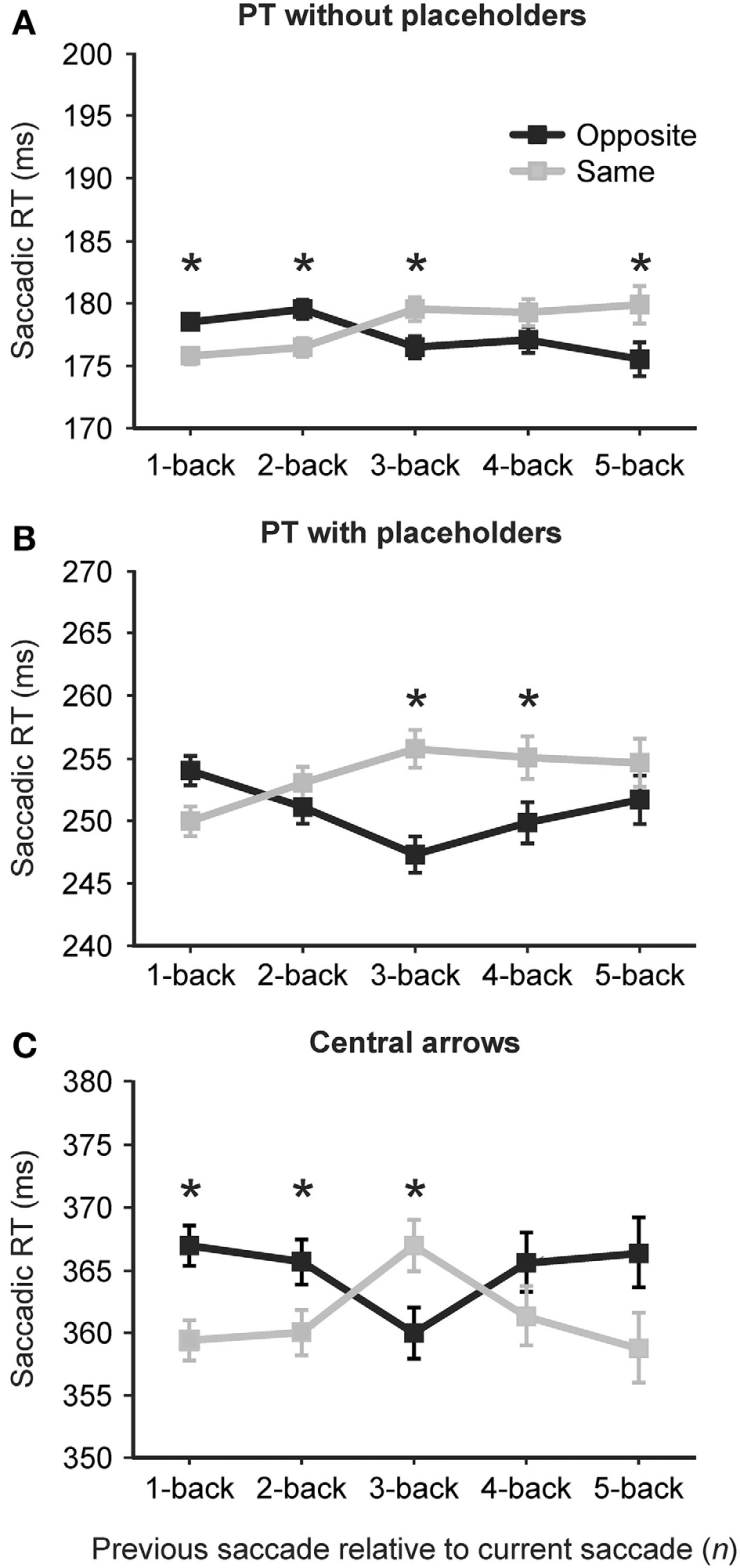
**Average saccadic reaction time (in ms) for each of the peripheral target (PT): without placeholders (A), peripheral target (PT): with placeholders (B) and central arrow conditions (C) as a function of previous saccade direction relative to the current saccade [same direction as the current saccade (light gray lines and symbols) or opposite direction as the current saccade (black lines and symbols)] and the number of intervening saccades [*1-back* (0 intervening saccades) to *5-back* (4 intervening saccades)]**. Error bars are standard error of the mean. Asterisks indicate a significant difference in reaction time between same and opposite.

Figure 3 shows average saccadic reaction times for each of the PT without placeholders (A), PT with placeholders (B) and central arrow conditions (C) as a function of previous saccade direction relative to the current saccade [same direction as the current saccade (light gray lines and symbols) or opposite direction as the current saccade (black lines and symbols)] and the number of intervening saccades [*1-back* (0 intervening saccades) to *5-back* (4 intervening saccades in any direction)]. Error bars are standard error of the mean. A summary of mean reaction times (in ms) for each saccade direction relative to the current saccade (same vs. opposite) as well as the *F* values, *p*-values and effect sizes (Cohen's d) for these comparison are presented in Table 2. Overall, reaction times varied across the three conditions [*F*_(1.75, 43.74)_ = 399.03, *p* < 0.001], with significantly faster reaction times observed in the PT without placeholders condition (*M* = 180 ms, *SD* = 19 ms) than the PT with placeholders condition (*M* = 259, *SD* = 28) than the central arrow condition (*M* = 372, *SD* = 38).

**Table 2:** **Average saccadic reaction times (in ms), *F*-values, *p*-values and Cohen's *d* for each n-back comparison between same and opposite trial types (alpha = 0.05)^†^**.

	***n−1***	***n−2***	***n−3***	***n−4***	***n− 5***
**PERIPHERAL TARGETS: WITHOUT PLACEHOLDERS**					
Same RT (in ms)	175	176	179	179	179
Opposite RT (in ms)	178	179	176	177	175
F-statistic	5.78	4.75	5.81	2.23	4.21
*p*-value	**0.024**	**0.038**	**0.021**	0.14	**0.044**
Cohen's d	0.07	0.09	0.03	0.06	0.09
**PERIPHERAL TARGETS: WITH PLACEHOLDERS**					
Same RT (in ms)	249	252	255	255	254
Opposite RT (in ms)	253	251	247	249	251
F-statistic	3.44	0.474	17.4	4.88	1
*p*-value	0.075	0.49	**<0.001**	**0.036**	0.32
Cohen's d	0.08	0.02	0.14	0.08	0.08
**CENTRAL ARROWS**					
Same RT (in ms)	359	360	367	361	358
Opposite RT (in ms)	367	365	360	365	366
F-statistic	6.29	10.64	4.9	2.26	2.53
*p*-value	**0.019**	**0.003**	**0.035**	0.14	0.12
Cohen's d	0.11	0.05	0.08	0.03	0.08

† *Boldface type indicates significance at the p < 0.05 level*.

### The effect of the immediately preceding saccade (n−1)

Analysis revealed an effect of the direction of the immediately preceding (*n−1*) saccade on current saccade latency in the PT without placeholders [2A, same: 175 ms, opposite: 178 ms, *F*_(1, 26.11)_ = 5.78, *p* = 0.024] and central arrow conditions only [2C, same: 359 ms, opposite: 367 ms, *F*_(1, 25)_ = 6.29, *p* = 0.019, Table 2]. In particular, in both of these conditions, saccadic reaction times were faster when the immediately preceding saccade (*n−1*) was in the same direction as the current saccade *n* (i.e., LL or RR) or a “same direction benefit” (SDB). A non-significant SDB was found in the PT with placeholders condition [2B, same: 249 ms, opposite: 253 ms, *F*_(1, 25.21)_ = 3.44, *p* = 0.075]. For greater *n-back* levels, this pattern diverged between conditions (as summarized below).

### The effect of previous saccades (n−2 through n−5)

In the PT without placeholders condition, the SDB observed at the *n−1* level persisted at the *n−2* level [same: 176 ms, opposite: 179 ms,; *F*_(1, 28.45)_ = 4.75, *p* = 0.038], but was replaced by a significant opposite direction benefit (ODB) at the *n−3* and *n−5* levels (Figure 3A, Table 2, no statistically significant difference between same and opposite at the *n−4* level). In the PT with placeholders condition, a significant ODB was found at the *n−3* and *n−4* levels (no difference between same and opposite at the *n−2* or *n−5* levels although a trend toward an ODB at these *n-back* levels, Table 2). In the central arrow condition, a significant SDB was revealed at the *n−1, n−2*, and *n−5* levels (non-significant SDB at the *n−4* level), with a significant ODB found at the *n−3* level (Table 2 and Figure 3C).

## Discussion

We adapted Anderson et al.'s (2008) random walk sequential saccade paradigm to examine the effects of preceding eye movements on saccadic latency in three stimulus conditions: PT without placeholders (as employed by Anderson et al., 2008), PT with placeholders (in which all possible target locations remained present during the entirety of the testing session), and central arrows (with placeholders). Overall, reaction times were fastest in the PT without placeholders condition and slowest in the central arrow condition. When examining the effect of the immediately preceding saccade on current saccade latency, we found that in both the PT without placeholders and central arrow conditions, saccadic reaction times were faster when the immediately preceding saccade was in the same direction as the current saccade. This pattern did not persist across all n-back levels and diverged between conditions. Ours is the second paper to employ the random walk paradigm as a method for examining directional relationships among saccades and the first to explore the effects of placeholders and central arrow cues on the interactions between current and prior saccades.

### Stimulus and saccade history effects do not simply diminish over time

Consistent with Anderson et al. (2008), we found significantly faster saccadic reaction times when the immediately preceding saccade (*n−1*) was in the same direction as the current saccade in our PT without placeholders condition (same: 175 ms vs. opposite: 178 ms) or a SDB. Additionally, we have extended this result to a new random walk task: the central arrow condition (Central arrows: 359 vs. 367 ms). Although the effect of previous saccade direction on current saccade latency in the PT with placeholders condition was not statistically significant at the *n−1* level, a similar trend was found in this condition. We will begin by discussing our results as related to our previously presented scenarios about the effects of saccade *n−1* on saccade *n* (hypotheses presented in Section Analyses and Hypotheses and Figure 2).

#### Stimulus and saccade history effects as a product of the execution of eye movements

We first considered the possibility that the *n−x* effects on saccade *n* latency presented by Anderson et al. (2008) might have arose due to the interactions related to the execution of eye movements in general, as opposed to repeated retinal stimulation due to peripheral targets on the opposite direction trials (Anderson et al., 2008). For example, in their exploration of saccadic dependencies in a real world visual search paradigm, Smith and Henderson (2011) suggested that the increase in saccade latency for two saccades to the same location might be a large part due to an overall bias that we have to direct saccades in the same direction (i.e., saccadic momentum), as opposed to only the avoidance of a previously fixated location. Saccadic momentum effects, whereby participants are biased to continue to saccade in the same direction, have been found in a number of visual search tasks including free (e.g., MacInnes et al., 2014) and array-defined visual search tasks (Hooge and Erkelens, 1996; Hooge and Frens, 2000). However, if this were the case in the random walk paradigm, we would have expected an extension of the SDB observed by Anderson et al. to the two new conditions we employed (PT with placeholders and central arrows). We failed to find a significant SDB at any *n−x* level in our PT with placeholders condition and the SDB observed in the PT without placeholders and central arrow conditions did not persist across *n−x* levels. This result suggests that the result reported by Anderson et al. (2008) is not due to interactions related to the execution of eye movements in general.

It is possible that the nature of the task or targets employed (e.g., salience, separation between targets) play a role in saccade direction biases such as those reported by saccadic momentum. In fact, research has substantiated the existence of “gradients of importance” across parts of real world targets, such that a bias to return to a previously inspected target or part of a target is dependent on the complexity and functional importance of the target (e.g., Wilming et al., 2013). For example, visually inspecting a coffee mug might result in a bias to return gaze repeatedly to the handle because the handle guides physical interaction with the mug. As such, unlike many objects that are visually inspected in the real-world, the targets employed in the current study do not have functional use and so there is no pre-determined component of the target that might draw gaze more than any other component (e.g., the handle of a tool, the opening of a vessel etc.). Likewise, unlike, the tasks reported by Hooge and colleagues and MacInnes et al. (2014), the random walk paradigm might not elicit location-dependent gradients in salience because it is not self-paced or free visual search.

#### Stimulus and saccade history effects as a product of peripheral onset targets

A second possibility that was considered was that the SDB reported by Anderson et al. arose from a sensory mechanism related to the different retinal locations stimulated by peripheral targets in the “opposite direction” as compared to “same direction” sequences. This scenario would predict that a SDB would be observed in the two PT conditions only, but not the central arrow condition (note the flat light gray line in Figure 2B). While our results did not substantiate this possibility completely, we are not willing to completely dismiss this explanation either.

We found differences in the overall reaction times across the three conditions, with the shortest saccade latencies observed in the PT without placeholders condition and the longest latency observed in the central arrow condition. This difference in saccadic latency across conditions is reasonable as peripheral targets elicit more reflexive saccadic responses, responses that are likely to be faster than those elicited using central arrows (Abrams and Dobkin, 1994; Taylor and Klein, 2000; Fischer et al., 2003; Hilchey et al., 2012; Cowper-Smith et al., 2013). However, not only did we find differences in the overall reaction times across our three conditions, we also found differences in the patterns of effects of previous saccades on current saccade latency as a function of stimulus condition. Overall, these results provide some support for the presence of different mechanisms operating for peripheral and centrally presented targets.

For example, In the IOR literature, central arrows have been used to distinguish between effects that might arise from sensory vs. motor processes (Abrams and Dobkin, 1994; Taylor and Klein, 2000; Fischer et al., 2003; Hilchey et al., 2012; Cowper-Smith et al., 2013). It has been suggested that peripheral cues might elicit sensory effects due to repeated stimulation in the same retinal location and motor effects due to eye movement initiation, whereas central arrows are likely to elicit motor-related effects without the confound of repeated retinal stimulation. Although, based on the current literature, it is still unclear as to whether or not there are meaningful differences in the nature of saccadic dependencies that occur for saccades in response to centrally vs. peripherally presented targets. Abrams and Dobkin's (1994) results suggested that peripheral targets might result in additive sensory and motor contributions, based on the smaller effects observed for saccades guided by central arrows (in which a motor but not sensory contribution is possible) vs. peripheral targets (in which both sensory and motor contributions are possible). In contrast, Taylor and Klein (2000) found similar IOR for saccades to peripheral targets and central arrows. Hilchey et al. (2012) reconciled these disparate findings by demonstrating that differences between central and peripheral targets arise only when stimulus conditions are blocked (i.e., participants complete all trials with peripheral targets, followed by all trials for the central target type), and are therefore likely driven by attentional control settings related to the processing of peripheral cues rather than by differences in the nature of the effect occurring for peripheral and central targets. Peripheral and central arrow cues were blocked in the current study. To date, we are the first to examine the use of centrally presented cues in a random walk paradigm.

#### Stimulus and saccade history effects as inhibition of return

In our last hypothesis, we considered the relevance of the *n−1* analysis to the phenomenon of inhibition of return. Oculomotor IOR has been proposed to promote efficient visual search behavior by reducing the likelihood of revisiting previously searched locations (Klein, 1988; Klein and MacInnes, 1999). In real world visual search—one could imagine a foraging scenario in which food needs to be found for survival—discouraging the return of gaze to old locations would increase the efficiency of visual search and may increase the likelihood that the target of interest would be found. If the SDB is distinct from the IOR phenomenon, then we might expect it to be observed whether or not possible target locations remain persistently visible during the task. In contrast, IOR is moderated by whether target placeholders are present (Klein, 1988; Klein and MacInnes, 1999; Birmingham and Pratt, 2005). So, if the SDB is related to IOR, then we might expect the SDB to also be moderated by the presence or absence of placeholders such that the absence of placeholders (as in our PT without placeholders condition) should result in a smaller SDB than the two conditions in which placeholders are employed. While the magnitude of the SDB was greater in the central arrow (with placeholders) than the PT without placeholders condition, we did not observe the SDB in the PT with placeholders condition so we cannot completely substantiate this explanation.

The effects of previous saccade direction observed in the present investigation—and those reported by Anderson et al. (2008)—are smaller than IOR that has been previously reported in target-target saccade paradigms with the predictable return to center movement in between target presentations. For example, Taylor and Klein (2000) reported 21 ms of IOR in their saccade-saccade condition with peripheral targets, which included placeholders (vs. 4 ms in the PT with placeholders condition here) and 21 ms of IOR in their saccade-saccade condition with central arrows (vs. 8 ms in our central arrow condition). Superficially, the relatively small “IOR” observed for IOR-like sequences occurring by chance in the context of an entirely random sequence of left/right target directions in the present study might suggest that the predictability of the return to center saccade in typically employed IOR paradigms might be an important contributor to the IOR phenomenon. A direct comparison of predictable and unpredictable return to center sequences is necessary before reaching this conclusion; after all, target separation was dramatically different in the present study (2.7°) and Taylor and Klein (2000) (7.9°) among other methodological differences related to stimulus timing and location within the display.

Overall, our results suggest that the interactions between prior and current eye movements are complex and may not conform to the argument that saccade history effects diminish over time (Anderson et al., 2008). Regardless, as objects in the environment do not disappear, the maintenance of placeholders in our peripheral target with placeholders condition is more ecologically valid than ours or Anderson et al.'s (2008) PT without placeholders conditions.

It is worth noting that Anderson et al. (2008) used a single-subjects design involving only a small number of participants (*n* = 3, the two authors of the study and one naïve participant), a large number of trials per participant (12,000–24,000 saccades), and single subject statistical analysis (which is more sensitive to individual differences). By comparison, most studies exploring saccadic interactions employ a group approach with many participants (often 12 or more), a relatively small number of trials (usually less than 300), and within-subject or mixed analysis of variance (i.e., ANOVA). Because single subject designs rely on many repeated measurements of the variable(s) of interest over a longer period of time, they can better detect the true pattern and magnitude of the effects of interest, while simultaneously accounting for variations in individual participant behavior that might influence the effects (c.f. Gravetter and Frozano, 2012, pp. 395–430). It is possible that the SDB observed in the single-subjects design is not sufficiently robust to be detectable using a group design. However, in the absence of a direct replication of Anderson et al.'s (2008) results using their single subject analysis, we cannot draw this conclusion.

### Other effects of stimulus and saccade history

#### Reduced latencies for repeated locations

Munoz and colleagues (Dorris et al., 1999; Gore et al., 2002) have reported shorter saccadic latencies when gaze is brought to the same location repeatedly—an effect that appears to be opposite to that reported by the oculomotor inhibition of return literature. While these effects could be a product of involving non-human primates as test subjects, somewhat consistent with this finding is the significant ODB that we found at higher n-back levels. Together, these results might suggest that there is a time-dependent effect of the influence of previous saccades (*n−x*) on saccade *n* latency (e.g., perhaps due to residual neuronal activation due to previously executed saccades). In fact, Gore et al. (2002) reported a non-significant decrease in the benefit observed for two consecutive saccades to the same location as the inter-trial interval (time in between the first and second saccade) increased. Future studies employing the random walk paradigm might benefit from varying the time course of the elicitation of saccades, perhaps identifying instances of inhibitory and/or facilitative effects of previous saccades on current saccade latencies.

#### Current fixation location

**Figure 4:**
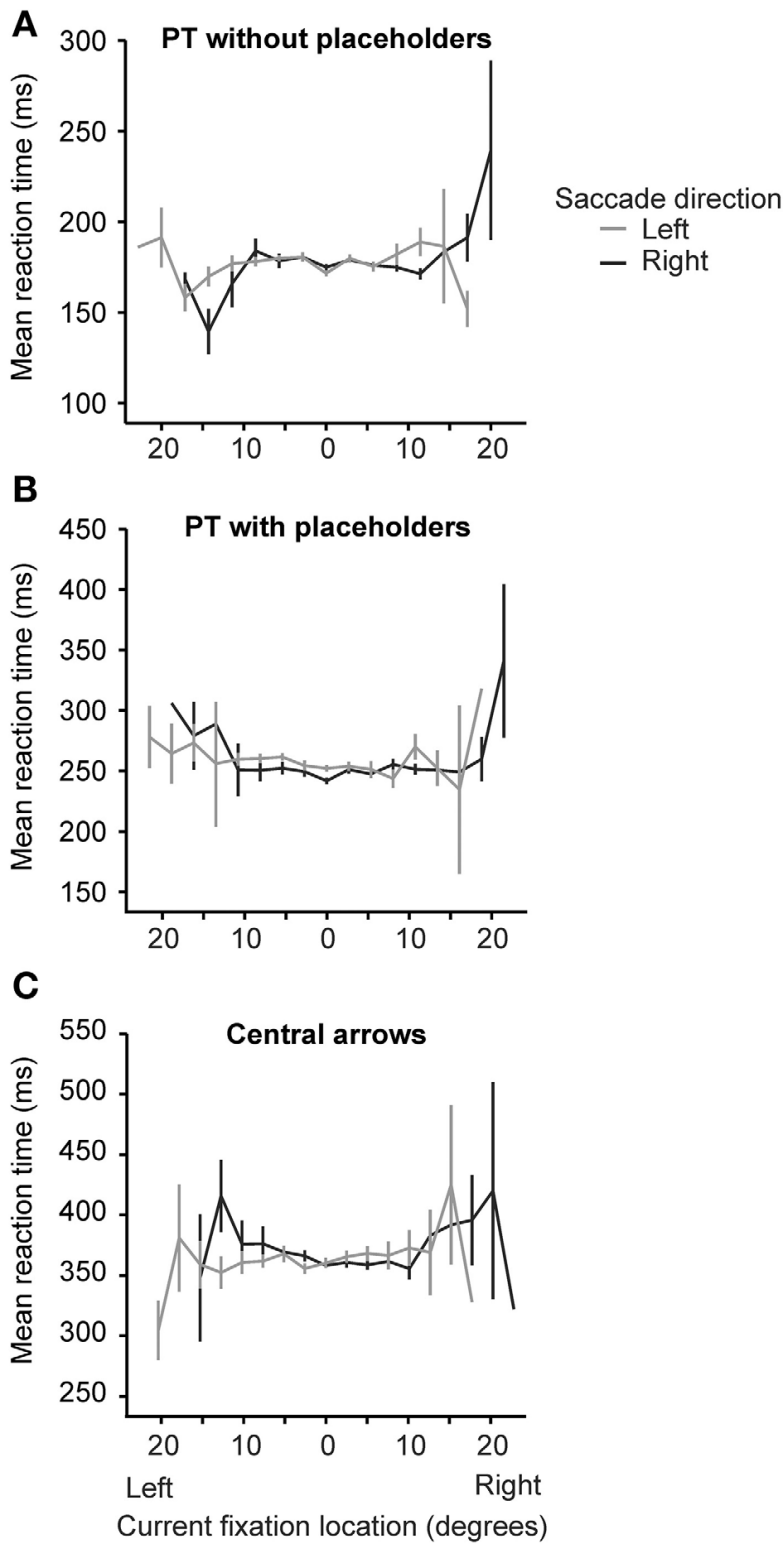
**Average reaction time (in ms) as a function of current fixation location eccentricity (in degrees) and saccade direction (left: gray lines or right: black lines) for each of the three conditions [peripheral target without placeholders (A), peripheral target with placeholders (B) and central arrow (C)]**. Error bars are standard error of the mean.

We considered the possibility that current fixation location (i.e., screen location) may co-vary with saccade direction to affect saccade latency. In particular, the possibility that, as subsequent saccades bring gaze to more eccentric locations (far left or far right), that participants may *expect* a subsequent saccade to be cued in the opposite direction (despite the random nature of the random walk paradigm). In this case, we might expect faster leftward saccades when current fixation location was farther right and faster rightward saccades when current fixation location was farther left. Figure 4 plots average reaction time as a function of current fixation location eccentricity and saccade direction (left or right). When we regressed reaction time on current fixation location (19 possible locations) and the interaction between current fixation location and saccade direction (2 directions; 38 cells in total) within each of the three conditions, we found no significant interactions between current fixation location and saccade direction in our PT without placeholders [*t*_(3395)_ = −0.09, *p* = 0.93, β = −0.002] and central arrow conditions [*t*_(3845)_ = −0.504, *p* = 0.61, β = −0.009]. While the interaction between current fixation location and saccade direction was significant in the PT with placeholders condition [*t*_(3968)_ = −2.45, *p* = 0.014, β = −0.04], the pattern of results do not suggest that as current fixation location became more eccentric, participants anticipated a cue to saccade in the opposite direction. In particular, saccades to the left beginning from eccentric rightward locations were not faster than right saccades from the same locations (likewise for right saccades from eccentric leftward locations), as would be expected if participants were anticipating cues to direct subsequent saccades toward the center (c.f. Figure 4). These results are consistent with those reported by Anderson et al. (2008, Figure 4 in particular).

#### Other potential effects

Similar to the consideration of current fixation location described in the previous section, biases similar to “gambler's fallacy” might arise in the current paradigm. For example, participants might decide that the probability of a target being presented to the left of their current fixation location is higher following several sequential presentations of rightward targets (or vice versa). Such biases could operate at any of the *n−x* levels, but the strength of the effect would be relatively small for small *n−x* levels and larger for greater *n−x* levels (e.g., like flipping a coin, the statistically ignorant gambler begins to expect a “head” outcome only after a relative large number of “tail” outcomes in a row). As such, the potential contribution of a gambler's fallacy to the current study is likely to be relatively minor. Further to this, there are relatively few sequences in our data for which all *n−x* saccades are in the same direction (of course this is more pronounced for longer sequences) and an analysis of the gambler's fallacy would be (necessarily) confounded by the potential accumulation of “same direction” effects. In other words, even if one were to demonstrate that a “different” saccade had a reduced reaction time relative to a “same” saccade after a sequence of “same” saccades, one could not say for certain that this was the result of incorrect participant expectations about the likelihood of a particular saccade direction, or if it was the result of an accumulation of effects due to the previous saccades themselves. Future research might benefit from an investigation of this question.

Although our criterion for correct saccades was appropriate for our task, we accepted saccades that terminated outside the perimeter of our targets as correct. It is, therefore, possible that participants made corrective saccades following their initial saccades as a way to return their gaze to a more central portion of the target. We did not quantify the prevalence of such corrective saccades, nor did we consider any effect they might have on subsequent saccade latencies. An inspection of the visual representations of saccadic history (video) for a subset of participants in our study suggests that while corrective saccades were possible, they were infrequent and within the boundary of the target.

Lastly, we must consider that we have assumed that the effects of “same” and “opposite” trial sequences on saccade *n* latency are approximately equal. In the absence of a baseline to which the “same” and “opposite” conditions can be compared, our methods and analysis permit only a comparison *between* “same” and “opposite” trials and do not allow us to determine if the magnitude of the effect of “same” and “opposite” on saccade *n* latency was equivalent.

### Summary and conclusions

The random walk sequential saccade paradigm (Anderson et al., 2008) permits an exploration of the influence of prior saccades on current eye movements, avoiding the potential pitfalls associated with the use of a central fixation location to which gaze (and attention) is drawn after saccades to targets. Here, we extended the random walk paradigm to: (1) examine the role of visual placeholders in saccade history effects in the random walk paradigm with peripheral targets; and (2) compare saccade history effects in peripheral and central stimulus conditions which differ in sensory but not motor characteristics.

We identified small but statistically reliable previous saccade effects at many *n-back* levels. At the *n−1* level, these effects are broadly consistent with the presence of oculomotor IOR, revealing that saccades had longer latencies when previous saccades were in the opposite direction, as would occur when revisiting a previously inspected target location. IOR is known to be reduced or eliminated when stable visual references are eliminated (Klein, 1988; Klein and MacInnes, 1999; Birmingham and Pratt, 2005), a result not apparent in our data. In all conditions, an ODB emerged at one or more of the higher *n-back* levels, indicating that there might be a time-dependent effect of previous saccade history on saccade *n* latencies. The present results also indicate some differences between central and peripheral target conditions, consistent with the possibility that interactions between prior and current saccades are likely due to multiple sensory and motor mechanisms. Overall, our results suggest that sequential saccade effects could be due to multiple, time-varying mechanisms related to sensory (i.e., retinotopic stimulus location), motor (i.e., saccade direction), and environmental (i.e., persistent visual objects) aspects of the task structure. Further research is needed to distinguish between these possibilities.

### Conflict of interest statement

The authors declare that the research was conducted in the absence of any commercial or financial relationships that could be construed as a potential conflict of interest.
